# Investigating two consecutive catastrophic breeding seasons in a large king penguin colony

**DOI:** 10.1038/s41598-023-40123-7

**Published:** 2023-08-10

**Authors:** Émile Brisson-Curadeau, Annette Scheffer, Phil Trathan, Fabien Roquet, Cédric Cotté, Karine Delord, Christophe Barbraud, Kyle Elliott, Charles-André Bost

**Affiliations:** 1 UMR 7372-CNRS, Centre d’Études Biologiques de Chizé, La Rochelle University, Villiers-en-Bois, France; 2 Natural Resource Sciences, McGill University, Sainte-Anne-de-Bellevue, QC Canada; 3 OKEANOS Centre, University of the Azores, 9901-862 Horta, Portugal; 4 British Antarctic Survey, High Cross, Madingley Road, Cambridge, CB3 0ET UK; 5 National Oceanography Centre, Waterfront Campus European Way, Southampton, SO14 3ZH UK; 6 Department of Marine Sciences, University of Gothenburg, Gothenburg, Sweden; 7 Laboratoire d’Océanographie et du Climat: Expérimentations et Approches Numériques (LOCEAN-IPSL), CNRS, IRD, MNHN, Sorbonne Université, Paris, France

**Keywords:** Behavioural ecology, Community ecology, Population dynamics

## Abstract

Large-scale breeding failures, such as offspring die-offs, can disproportionately impact wildlife populations that are characterized by a few large colonies. However, breeding monitoring—and thus investigations of such die-offs—is especially challenging in species with long reproductive cycles. We investigate two unresolved dramatic breeding failures that occurred in consecutive years (2009 and 2010) in a large king penguin *Aptenodytes patagonicus* colony, a long-lived species with a breeding cycle lasting over a year. Here we found that a single period, winter 2009, was likely responsible for the occurrence of breeding anomalies during both breeding seasons, suggesting that adults experienced poor foraging conditions at sea at that time. Following that unfavorable winter, the 2009 breeding cohort—who were entering the late stage of chick-rearing—immediately experienced high chick mortality. Meanwhile, the 2010 breeding cohort greatly delayed their arrival and egg laying, which would have otherwise started not long after the winter. The 2010 breeding season continued to display anomalies during the incubation and chick-rearing period, such as high abandonment rate, long foraging trips and eventually the death of all chicks in winter 2010. These anomalies could have resulted from either a domino-effect caused by the delayed laying, the continuation of poor foraging conditions, or both. This study provides an example of a large-scale catastrophic breeding failure and highlights the importance of the winter period on phenology and reproduction success for wildlife that breed in few large colonies.

## Introduction

Animals face extreme challenges during reproduction, such as poor foraging conditions, unfavorable weather, or diseases^1,2^. Occasionally, these challenges are simultaneously experienced by most individuals of a population, resulting in a large-scale breeding failure.

Perhaps the most impressive manifestations of breeding failures occur when massive die-offs of offspring are observed in colonial species. For example, heavy precipitation and extreme sea-ice conditions in 2014 led to all chicks dying at a 34,000 pair Adélie penguin *Pygoscelis adeliae* colony located in Antarctica^3,4^. Yet, the cause of such die-offs is not always readily identified, particularly in species breeding over long periods; monitoring throughout the full reproductive cycle is generally costly and logistically challenging.

The king penguin *Aptenodytes patagonicus* is one seabird species that has an unusually long breeding season: pairs take approximately 13–14 months to fledge a chick, one of the longest breeding cycles among birds^5^. Chick-rearing starts in the summer, but parents leave the colony for the winter, while the chick fasts unsupervised on land. In spring, adults come back to the colony to resume chick provisioning, while the next breeding wave has already started incubation. This long breeding cycle prevents most of the birds to lay annually, creating overlap between cohorts and complicating the understanding about large-scale breeding failures. In fact, this species has also suffered from large-scale chick die-offs in the past at several localities, some resulting from prey depletion, climate anomalies or even tsunamis^6–8^. Yet, some colony breeding failures remain unresolved, as the initial causes are particularly hard to determine over such a long breeding cycle^5^.

Here, we report and investigate two of the most catastrophic breeding failures in king penguins, which occured in a 100,000 breeding-pair colony on Kerguelen Island. During the consecutive breeding seasons of 2009 and 2010, two seemingly unrelated massive die-offs occurred among the chicks. We use the available tracking and diving data, chick monitoring and colony counts from the two breeding seasons for comparison with similar data from the long-term monitoring program at the colony available between 1998 and 2022. This allowed us to provide a complete description of the catastrophic failure with regards to the “normal” years and establish the chain of events that led to the breeding failures. Finally, we explore the possible triggers of the failures. Because there is negligible interaction between king penguins and fisheries in this region, we expected the failures to be driven by extreme environmental conditions affecting prey availability. We therefore explored the environmental variables that are know to affect foraging conditions and that could have affected each reproduction season.

## Results

### Breeding season 2009

Available data from 2009 included tracking and diving data during the chick-rearing period, as well as chick mass and survival monitoring (see “Methodology” section for further details).

During the early chick-rearing period (i.e. February, summer) of 2009, foraging effort appeared to be normal, as foraging trip distance, trip duration, and dives per day were not statistically different from the other years (Fig. S1). Similarly, the foraging success metrics (dive wiggles per day—a proxy for prey pursuit—and mass gained per day) were not statistically different than other years (Fig. S1).

**Figure 1:**
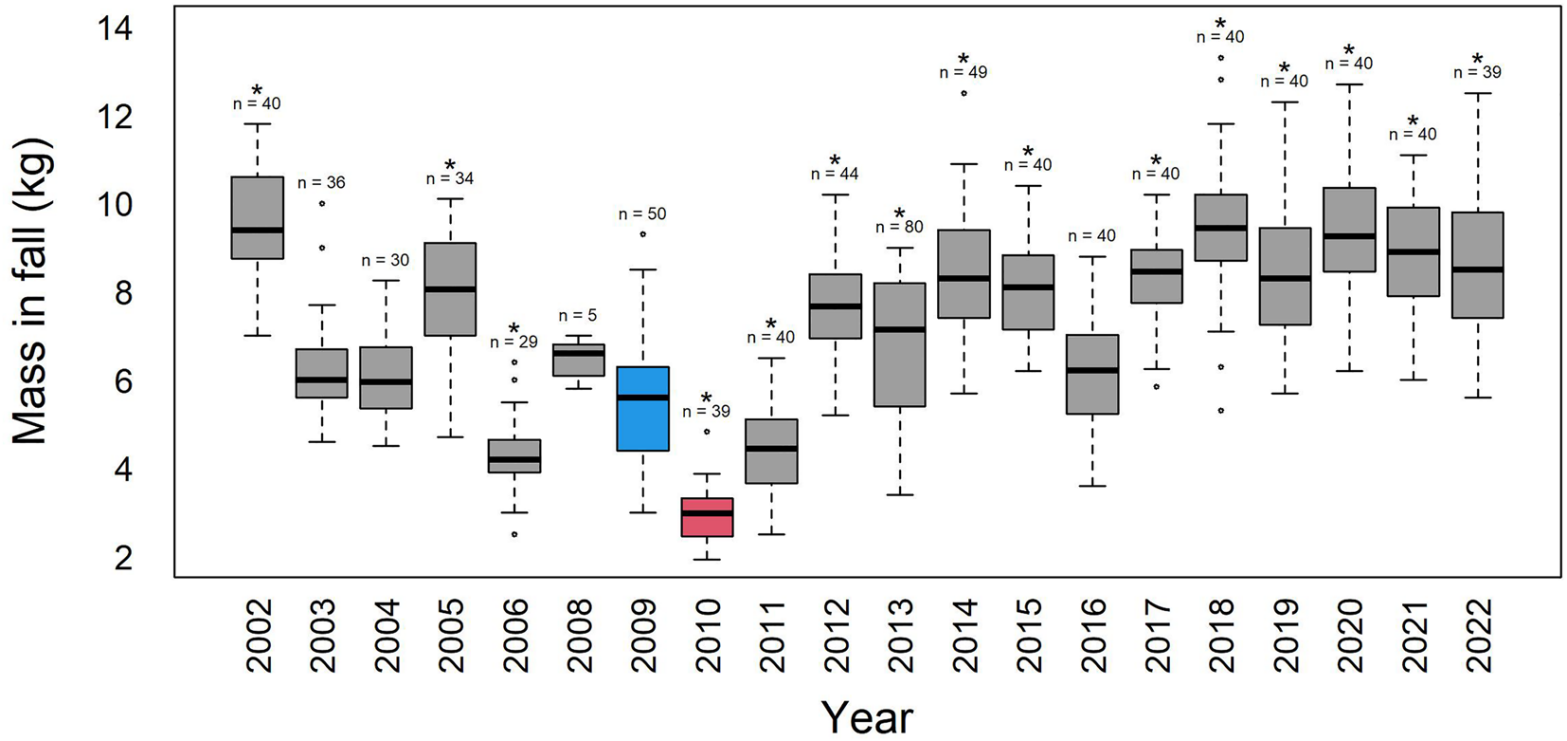
Chick survival throughout the winter. Results for the 2009 breeding season is in blue and results for the 2010 breeding season is in red. For a given period, survival rates that are significantly different than either 2009 or 2010 are represented with an asterisk (*), those that are significantly different than both years are represented with an eight-spoke star (⁕).

**Figure 2:**
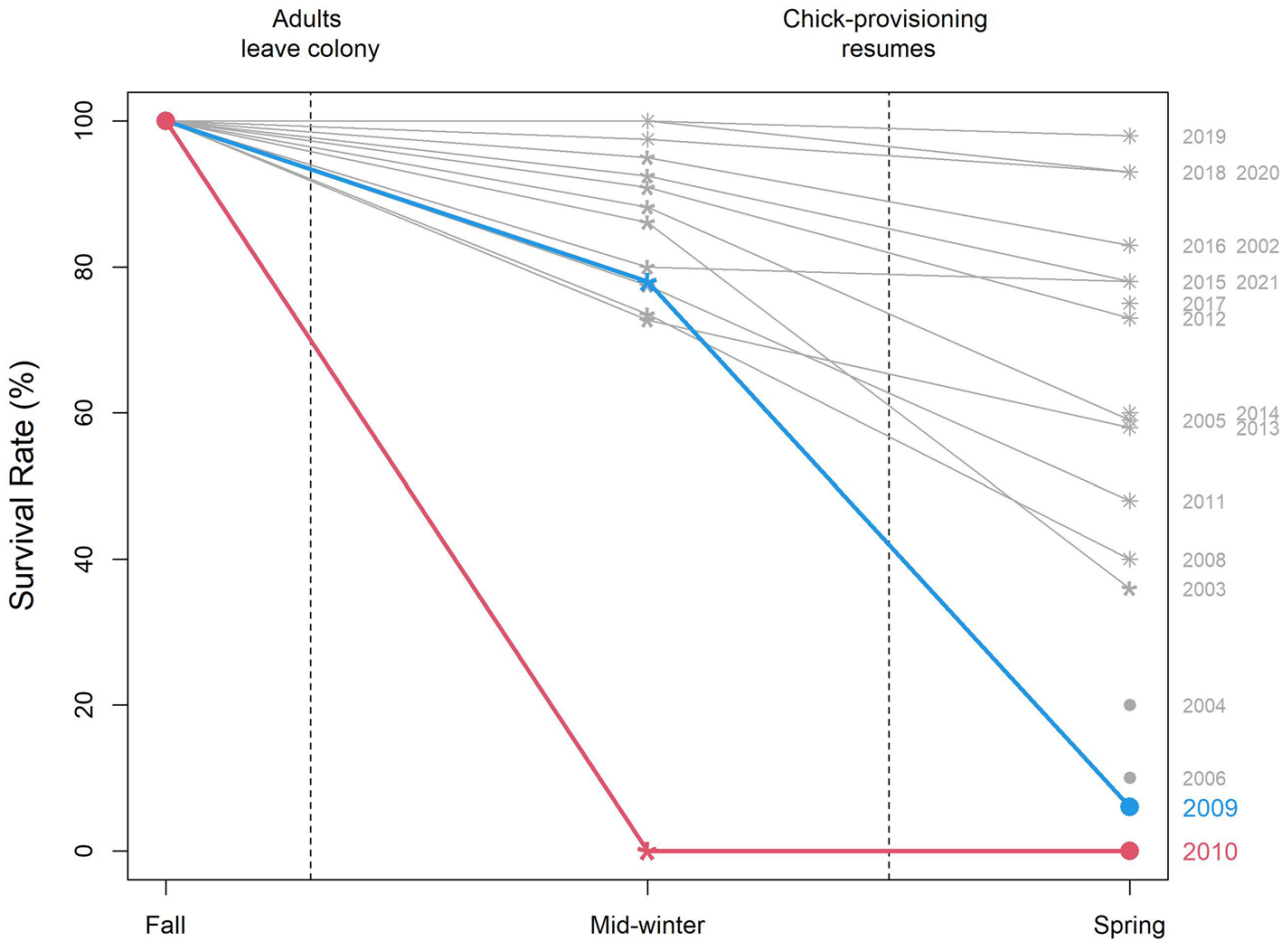
Chick mass in fall, before the winter fast. The 2009 breeding season is in blue and the 2010 breeding season is in red. Masses in all years are significantly different from 2010 (Tukey’s Test). Asterisks are displayed above years that are significantly different from 2009.

Chick mass before winter (i.e. April) in 2009 was lower than average, but still within the range of the observed variations: it was even significantly higher than 3 other years (Fig. 1). Similarly, chick survival in mid-winter (i.e. July) was 78%, which was below average but not exceptionally low, as it was only significantly lower than 3 of the 12 years with similar data (Fig. 2). In contrast, chick survival decreased drastically between mid-winter and the following spring (i.e. October), as survival decreased to 6%, which was lower than all other 17 years (not accounting for 2010) and significantly so for 14 of the 17 years (Fig. 2). The low yet not unprecedented breeding success of the three other years was due to low ocean productivity during the chick-rearing period and was explained in Brisson-Curadeau et al.^9^.

### Breeding season 2010

Available data from 2010 included colony counts, tracking and diving data during the incubation period, as well as chick mass and survival monitoring (see “Methodology” section for further details).

A marked delay in adult arrival was apparent during the 2010 breeding seasons. On December 14th 2009 (i.e. austral spring), when the 2010 parents were expected to have already laid their egg three weeks earlier, only 30 incubating pairs were counted in the entire colony. In comparison, the numbers were in the tens of thousands for similar dates in previous years (Table 1). While no official counts of incubating birds have been conducted since 2009, yearly visits at the colony in December from 2011 to 2022 have also showed estimates in the tens of thousands (pers. obs.).

**Table 1:** Number of king penguin incubating pairs during each breeding season at Ratmanoff colony, Kerguelen.

Breeding season	Date of count	Number of incubating pairs
1999	18-12-1998	106,583
2005	16-12-2004	105,490
2006	22-12-2005	98,042
2008	16-12-2007	52,671
2010	14-12-2009	30

Note that incubation starts in the calendar year prior to the rest of the reproduction cycle.

Adults eventually arrived and by February 2010 (i.e. austral summer) there was seemingly normal numbers in the colony, but due to logistic constraints, an exact breeding count could not be undertaken.

**Figure 3:**
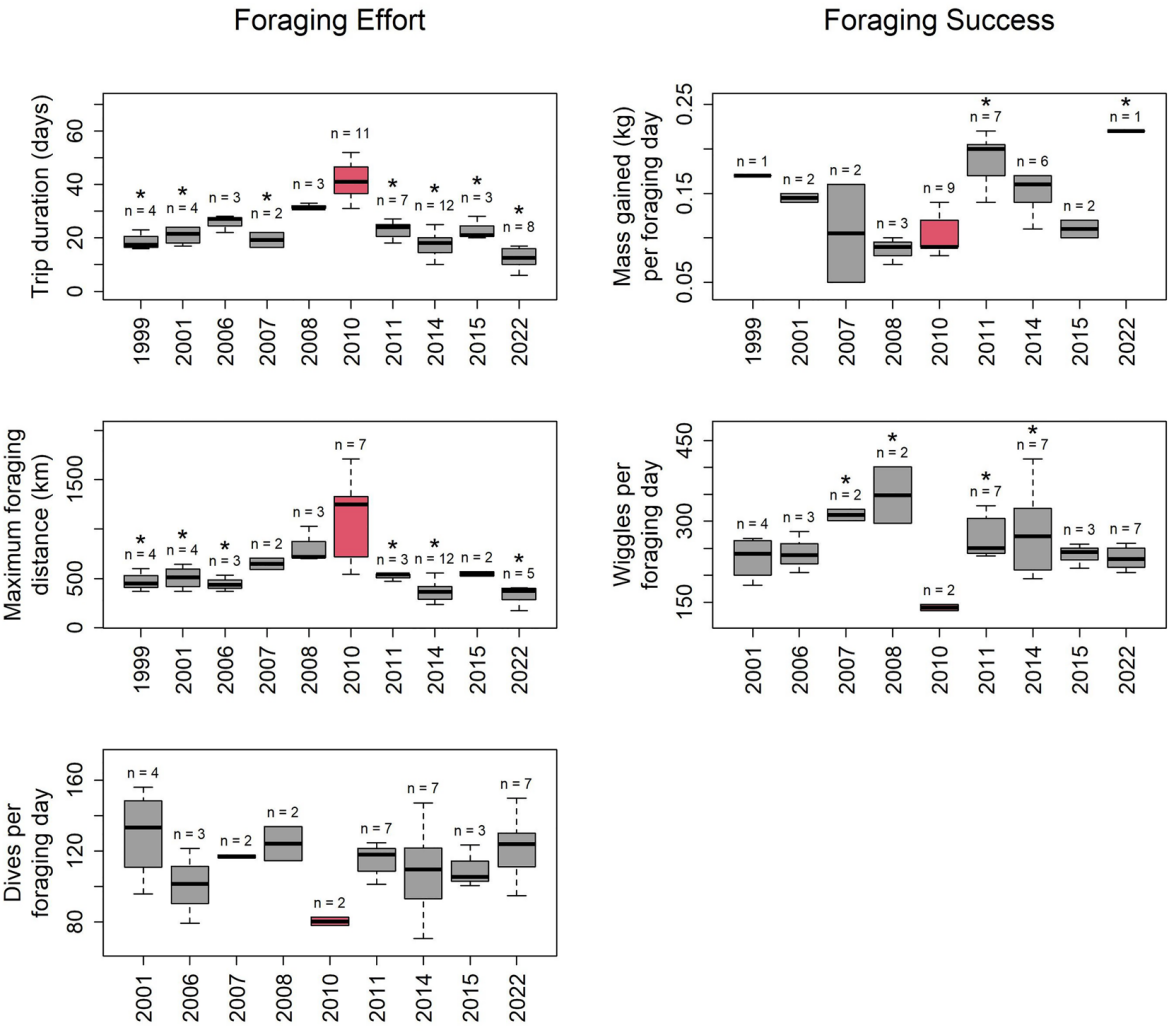
Foraging effort (left) and foraging success (right) variables during incubation. The year 2010 is highlighted in red. Asterisks are displayed above years that are significantly different from 2009 (Tukey’s Test).

**Figure 4:**
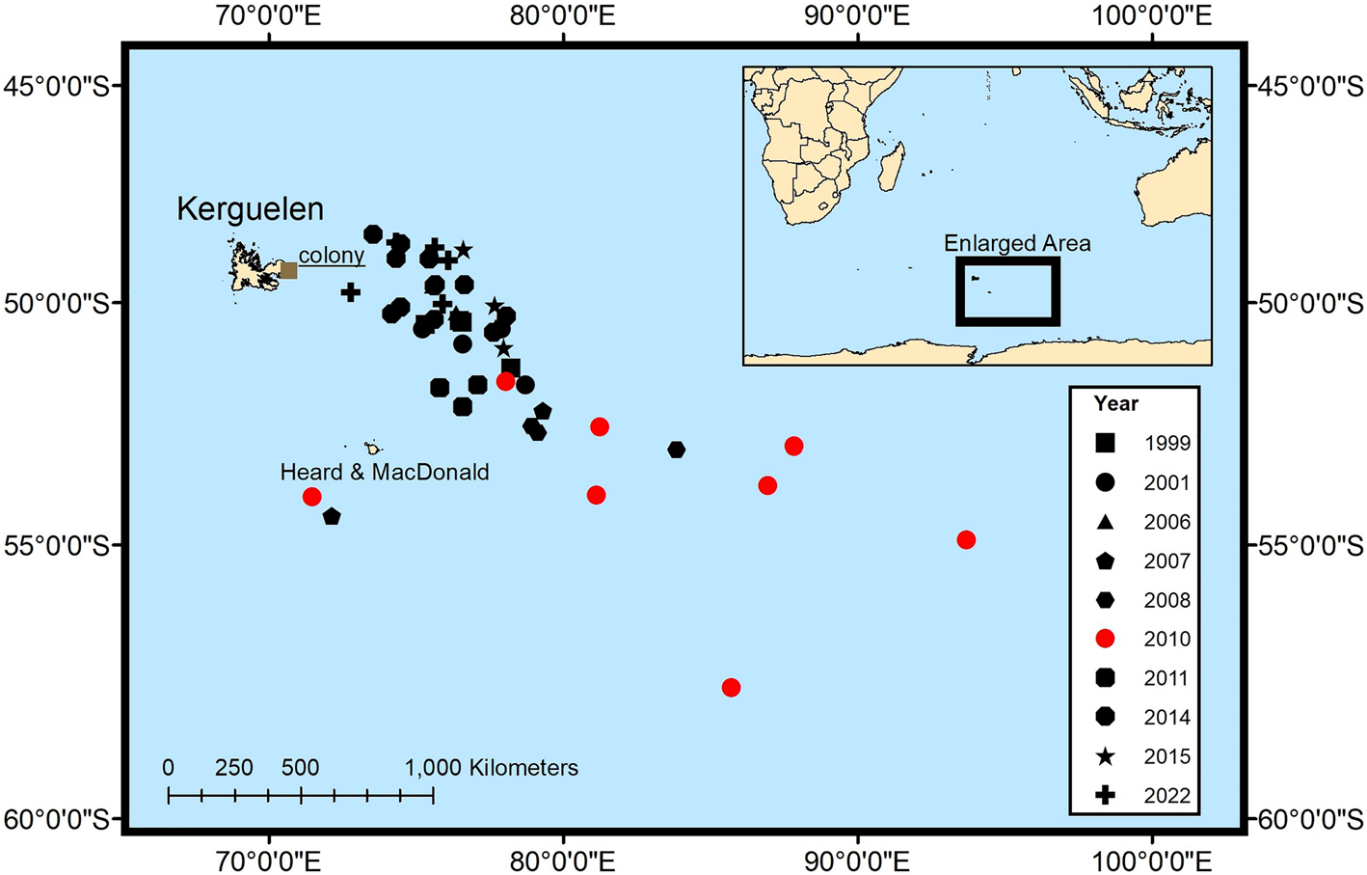
Furthest foraging location detected for each equipped penguin during incubation, sorted by year. In 2010, the foraging trips were significantly further from the other years. Map created using ArcGIS Pro 3.1. Country contour lines downloaded from http://tapiquen-sig.jimdo.com (Carlos Efraín Porto Tapiquén. Orogénesis Soluciones Geográficas. Porlamar, Venezuela 2015. Based on shapes from Enviromental Systems Research Institute. Free Distribution).

Egg abandonment rate during the incubation period was exceptionally high in 2010, with 93.8% of the 48 monitored pairs abandoning their egg. In contrast, none of the 85 monitored pairs over the eight other years with similar incubation data abandoned their egg (all years significantly different from 2010). Trip duration and foraging distance were longer in 2010 than all other 9 years with available incubation telemetry data (Figs. 3, 4). Dives per day and dive wiggles per day were both lower than all other years (Fig. 3), albeit with a very small sample size in 2010 because of a high desertion rate of tagged birds. Adult mass gained per foraging day was lower than average but not significantly different from six of the other eight years with similar data (Fig. 3).

The average chick mass before winter was much lower in 2010 (3.0 ± 0.6 kg) than the average (7.5 ± 2.0 kg), resulting in a significance difference when tested individually with all other years, including the 2009 cohort (Fig. 1). All monitored chicks died in the winter by July. The final chick survival rate of 0% was the lowest ever observed at this colony (Fig. 2) and in any large king penguin colony.

### Climate

**Figure 5:**
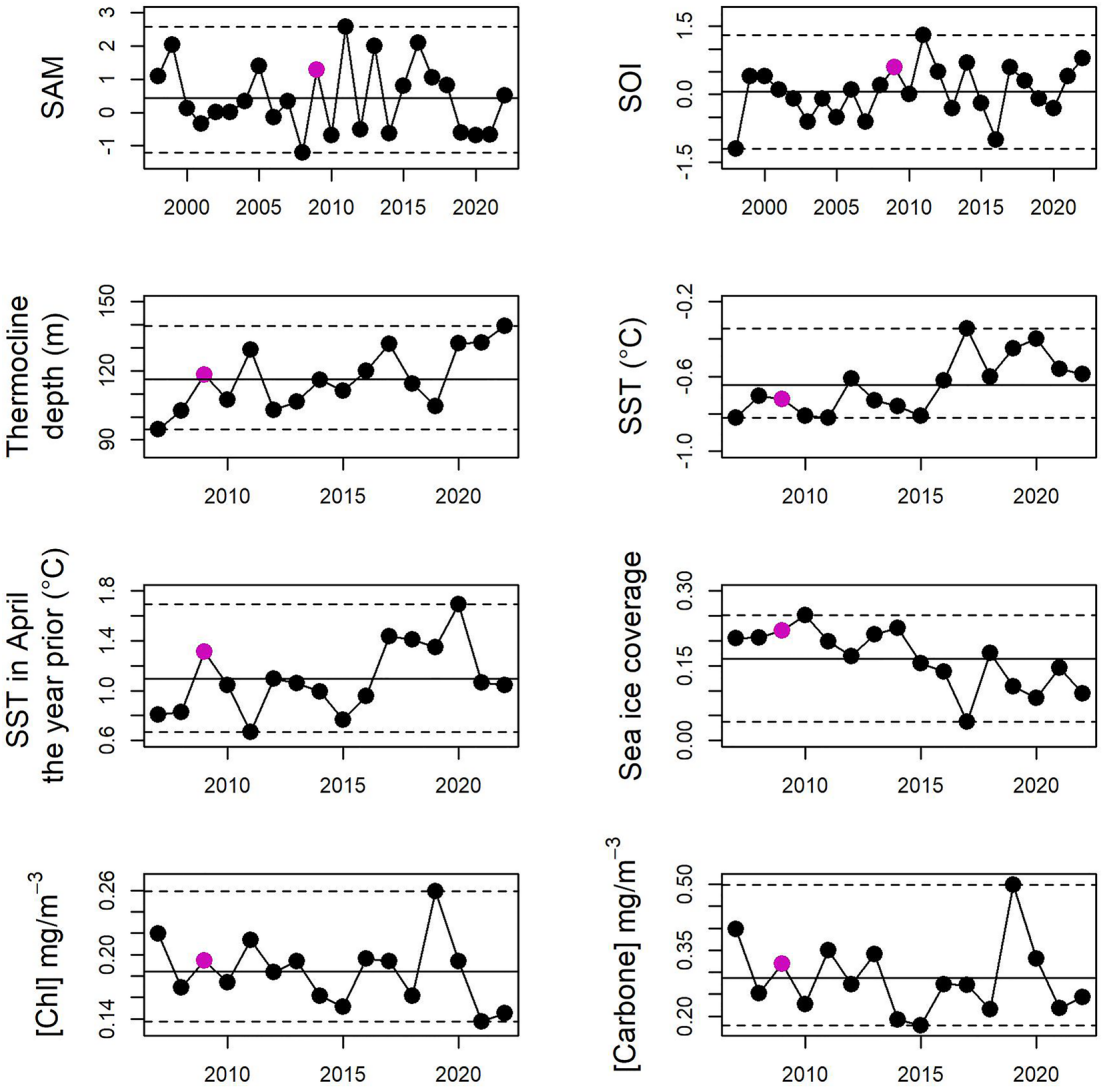
Climatic variables in winter (Jul–Aug averaged). The winter 2009—identified as the key period that triggered the breeding failure for both the 2009 and 2010 breeding seasons—does not show extreme values on any variables. Maximum and minimum values are represented by a dotted line.

From the results above, we postulated that the winter of 2009 led to the failures for both the 2009 and 2010 cohorts. Consequently, we explored whether any environmental variables showed extreme values during that period (average of July–August 2009). Sea ice coverage was extensive during that winter, but did not show the highest value of the dataset (Fig. 5). All other environmental variables showed approximately average values during the winter period.

Finally, it is noteworthy that all variables were average in February during both years, when foraging behavior was assessed (Fig. S2). Air temperatures at the colony during the winters 2009 and 2010 were also slightly below average (2009 =  − 0.22 °C below average, 2010 =  − 0.04 °C below average), but not extremely so compared to other years, such as 2005 (− 0.41 °C below average) and 2007 (− 0.37 °C below average).

## Discussion

**Figure 6:**
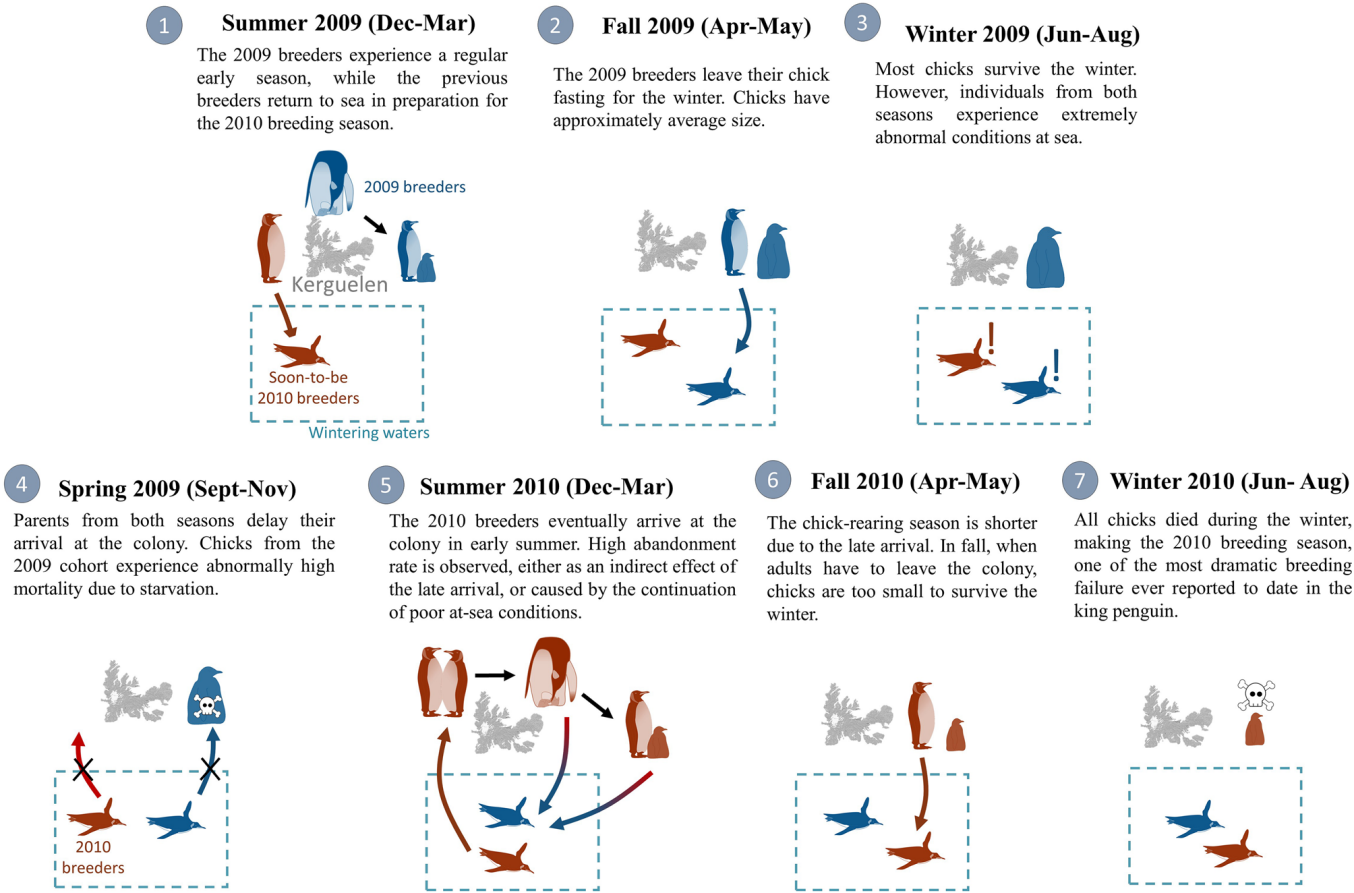
Proposed hypothesis retracing the events leading to the breeding failure in 2009 and 2010. The exact conditions during the winter 2009 that might have influenced the two reproductive seasons are still unknown.

We reported two catastrophic breeding failures occurring in a large colony of seabirds. During the years 2009 and 2010, we recorded the highest chick mortality documented for a large king penguin colony^10^. We suspect the winter 2009 to have been problematic for both breeding seasons, with adults likely encountering poor feeding conditions at sea, affecting chick provisioning during spring for the 2009 breeders and delaying egg laying for the 2010 breeders (see Fig. 6). For the 2010 breeders, it is unclear whether the problems encountered further in the reproductive season (egg abandonment, chick mortality, etc.) were caused by the continuation of these poor conditions, or by cascading effects resulting from delayed laying. Further questions around the 2009–2010 breeding failure at Kerguelen remain, as no environmental variables showed extreme values during the winter of 2009, leaving few clues as to what exactly could have caused the poor foraging conditions.

### The 2009 breeding season

The 2009 breeding season appeared unlikely to be catastrophic until the very end. Foraging success metrics during the chick-rearing stage were only slightly below average and well within the observed inter-annual variation. Similarly, chick mass before winter—a crucial determinant of chick survival over winter^9^—was low, but not unprecedented. Consequently, the survival of chicks in the middle of the winter was within the normal range.

However, almost all chicks had died by November, suggesting an important reversal by spring. When winter ends, adults usually come back to the colony and start feeding their chicks. For king penguins, the timing of the return of the adults in spring is critical, and any delay is thought to be fatal for the chick, which by the end of winter has low fat reserves^11^. We suggest that adults experienced very poor foraging conditions in winter (e.g. reduced prey availability), affecting their timing of return to the colony, with catastrophic consequences. Reports indeed suggest very low colony attendance as early as August (Chevalier, El Ksabi & Planade, pers. com.). This would confirm the delayed arrival of adults in early spring. Further continuation of poor foraging conditions into late spring could have also affected chick-provisioning rates.

### The 2010 breeding season

The 2009 low breeding success is second only to the 2010 breeding season, the most dramatic breeding failure recorded for king penguins. The first anomaly observed in this breeding seasons was the colony-wise > 3 week delay in egg laying. It is likely that this delay in arrival of adults was caused by the same factors which triggered the 2009 breeding failure. Indeed, during the winter 2009—when the 2009 breeders presumably started experiencing poor conditions at sea—the 2010 breeders were also at sea, accumulating reserves for the upcoming breeding season (see Fig. 6). In the following spring, both the 2009 and 2010 breeders experienced breeding anomalies: a chick-rearing failure for the 2009 breeders and a marked delay in egg-laying for the 2010 breeders. Therefore these two breeding seasons seem to have been affected by the same event, i.e. poor at-sea conditions in winter and/or early spring.

The breeding anomalies in 2010 never ceased and were recorded in other breeding stages; including unusually long trips at sea and high abandonment rates during the incubation period, as well as extremely low mass of chicks before the winter, and finally the death of all chicks in the winter.

We see two possible non-exclusive explanations for these breeding anomalies. Firstly, all these observations during the 2010 breeding season could ultimately be consequences from the delayed laying, passing on from one breeding stage to the other through cascading effects. Timing of life-history events is critical at the beginning of the season for king penguins, because time is limited for the chicks to gain sufficient mass to survive the winter fasting period^12^. For this reason, late arriving individuals may be less invested in their reproduction attempt, as the chances of success are low^5,13^. This might be what occurred in 2010, as the colony-wide delay in phenology led most birds to abandon their egg before it hatched. Parents that did not abandon their egg were less invested when foraging during incubation: they undertook long and distant foraging trips, while reducing diving effort. Indeed, individuals experiencing lower drive for breeding are not constrained by the need to return quickly to the colony, and so can undergo longer foraging trips^14^. Furthermore, they do not need to increase diving effort to sustain long fasting incubation shifts, contrary to birds more invested in breeding. By the onset of the winter, chick mass was less than half that of the long-term average, and by far the lowest ever recorded at that colony. This was likely caused by the short period available for penguins that year to provision their chick. The consequence was a massive winter die-off of chicks, unprecedented in a large king penguin colony.

Secondly, the poor conditions suspected to have taken place in winter 2009 could have continued during the following spring and perhaps summer. This also leads to a reduced parental investment and high abandonment rate. Furthermore, poor foraging conditions could partially explain the low mass of chicks before the winter, in addition to the delayed laying/hatching. Unfortunately, data on the foraging success during chick-rearing of 2010 are scarce because of the very low of successful tracked birds (48% return rate).

### Limitations and future directions

Examining causes behind anomalous events will always remain a difficult task because these events occur only rarely by definition, offering few replicates for investigation. The main unsolved issue around the 2009 and 2010 extreme breeding failures is the exact nature of the presumed poor conditions occurring in winter 2009. We did not find any relevant climatic variables that could explain a large-scale prey depletion during that period. Prey data (myctophid fish) is scarce in this region of the world, and foraging conditions for king penguin adults have usually been deduced indirectly from environmental variables, with a focus on the chick-rearing period^15–17^. More research is needed to fill the gap between climate, prey, and marine predators such as penguins, especially in winter. There is also a need to monitor the foraging success during all the breeding stages for pelagic foragers such as king penguins that are present year-round in the same oceanic region.

## Methodology

All data was collected at Ratmanoff’s colony in the Kerguelen archipelago (− 49° 14′ 33″, 70° 33′ 40″), situated in the southern Indian Ocean. The colony hosts nearly 100,000 breeding pairs of king penguins^18^. All experiments are in accordance with ARRIVE guidelines. Methods were carried out in accordance with the ethics committee of the Institut polaire français Paul-Émile Victor. The study was approved by the Ethics committee of Institut polaire français Paul-Émile Victor.

King penguins have an extended breeding cycle. Egg laying starts in the austral spring (late November), and hatching occurs 54–57 days later, in early summer, with a peak in late January^5,10^. Adults then start provisioning their offspring until the onset of winter, in mid-April, when they then leave the colony for up to 5 months, returning only occasionally to feed their offspring. Throughout winter, chicks fast and primarily survive on the fat reserves accumulated during the summer^11^. When adults return in spring (early September), they resume chick provisioning until December, when chicks finally fledge. At that point, the subsequent breeding season has already started. This extended chick rearing period implies that penguins can only successfully breed every other year^19,20^.

For simplicity, breeding seasons are named using the calendar year during which most of the reproduction cycle takes place. For example, birds of the “2010 breeding season” started incubation in November–December 2009, but provisioned their chicks throughout the summer 2010 (February to April), with a chick fasting period in winter 2010 (April to September), followed by another chick-provisioning period and fledging in spring 2010 (September to December).

The foraging range of the Kerguelen population during incubation and chick-rearing encompasses an area of 235,000 km^2^ immediately to the southeast of the colony (between 49° S and 53° S and 70° E and 78° E degrees^9,17^. In winter, adults migrate south to the northern limit of the seasonal sea ice (from − 55.7° to − 61.5° South and 57.6° to 79.8° East—Brisson-Curadeau & Bost, unpublished data, see Fig. S3).

### Colony counts during incubation

The colony was photographed with aerial images (either by helicopter or camera-fitted kites^21^ in mid-December during five years between 1998 and 2009. This period corresponds to the middle of the incubation stage, so that a photo taken in December 2009 will be associated with the 2010 breeding season. To estimate the number of breeding pairs, the images were stitched together and incubating penguins were counted by hand based on the typical incubation posture^21^.

### Foraging behavior during incubation

For the 2010 season, foraging data were obtained only during incubation, as high abandonment rates of tracked adults prevent the recovery of their data logger. It was then also decided to stop any telemetry of chick-rearing birds, given the breeding failure rates. Consequently, only foraging data during incubation were used to compare the 2010 breeding season with other years.

During nine years between 1999 and 2022, 44 individuals (including 7 in 2010) were equipped during incubation (December–February) with GPS or ARGOS tags from various manufacturers. From this data, the maximum foraging distance was calculated and used as a measure of foraging effort. During the same period, 37 individuals (two in 2010) were equipped with depth-loggers either from Wildlife Computer (Redmond, United States) or Technosmart (Colleverde, Italy). The number of foraging dives per day was calculated from this data and served as a metric of foraging effort. A foraging dive was defined as a dive with maximum depth > 50 m^22,23^. We also calculated the average number of undulations or wiggles in the dive per day as a measure of foraging success. Wiggles are short up-and-down motions in the depth-profile of a dive and are associated with prey capture attempts in king penguins^24–26^. Here, they are defined as an increase in the depth, followed by a decrease and another increase, with a minimum of 2 m in vertical deviation^25^.

All loggers were retrieved after the individuals had returned from their first trip to sea (typically lasting around 17 days). Birds were captured and weighed before and after the foraging trip to determine the mass gained per day at sea as another metric of foraging success, and trip duration was recorded as a measure of foraging effort. The breeding status of all equipped individuals were monitored during their foraging trip. If the incubating partner deserted the egg while the equipped individual was at sea, we considered the breeding status as failed. In the 2010 breeding season, when a high abandonment rate was apparent, an additional 37 breeding pairs from non-equipped individuals were monitored to better quantify incubation failure. In a similar fashion, 36 additional breeding pairs were monitored in 2011 for comparison.

The exact sample size for all years is reported in the supplementary material (Table S1), as well as in the results (Fig. 3). No data could be collected during incubation for the 2009 season. It is also noteworthy that egg abandon rate, trip length and mass gained per day can all have sample sizes slightly differing from the number of equipped individuals. Reasons explaining these occasional discordances are listed in the supplementary material (Table S2).

### Foraging behavior during chick-rearing

Foraging data in 2009 were only collected during the chick-rearing period (February 2009). Four individuals were equipped with an ARGOS tag, while thirteen were equipped with a depth-logger. Maximum distance from the colony, number of dives per day, and number of wiggles per day were calculated during this breeding period as well as for all others between 1998 and 2022, except for 2010 (i.e. 24 years, with a total of 161 GPS/ARGOS-equipped birds, 173 depth-logger-equipped birds). Mass gained per day (n = 380 individuals) and trip length (n = 583 individuals) were also recorded for those individuals as well as for additional non-equipped birds. The total sample size of equipped individuals for all years during chick-rearing is reported in the supplementary material (Table S3).

### Chick mass and survival

Chick mass and survival were assessed annually between 2002 and 2022, apart from 2007 (i.e. 20 study years). Randomly selected chicks were weighed and marked with fish tags (FloyTags, Seattle, USA) in April before the winter fast. The number of chicks marked varied between years, ranging from 28 to 55, with most years (15) having n = 40 chicks. The colony was visited in spring (October–November) to assess post-winter mortality of marked chicks. For 14 years, including the 2009 and 2010 cohort, mortality was also assessed earlier in July, corresponding to the mid-winter mortality.

### Environmental variables

To investigate if a climatic variable was responsible for the 2009 and 2010 breeding failures, we looked at variables known to affect the foraging and breeding of king penguins. At Kerguelen, the sea surface temperature in April with a 1 year lag (hereafter “SST_april-1 year_”) has been shown to be related to the breeding success of king penguins, and so was a plausible physical driver of these failures^9^. Four other probable drivers—chlorophyll concentration, thermocline depth, SST (no lag) and sea ice cover (in winter only)—have also been shown to relate to king penguins foraging at other colonies, and thus were also considered in our analyses^8,27–29^. The Southern Annular Mode (SAM) and Southern Oscillation Index (SOI), influence multiple components of ocean dynamics in both the wintering and breeding areas used for foraging, and so could potentially affect breeding success^30,31^. Furthermore, carbon concentration is an indicator of ocean productivity, and so was added as an additional plausible correlate. Finally, we looked at air temperature at the colony during the winter chick fast (June to August), as low temperatures been shown to negatively impact chick survival^9^.

Monthly multi-depth ocean temperatures, chlorophyll concentrations, carbon concentrations and Sea Ice Coverages (SIC) were obtained for the 2007–2022 period through the E.U. Copernicus Marine Environment Monitoring Service (CMEMS) at 0.25 geographic degree resolution^32^. Using the Global Ocean Reanalysis product, temperatures were retrieved from the 0 to 266 m range depth (28 depth points total), from which thermocline depth was calculated as the depth where temperature changes are maximal^33,34^. Monthly SAM^35^ and monthly SOI^36^ were obtained for the 1998 to 2022 period. Air temperature anomalies at the colony were obtained through the National Centers for Environmental Information^37^.

### Statistical analysis

We compared the across-year mean of each variables by building linear models with the variable year as the explanatory variable and the variable of interest (either foraging distance, trip durations, dives per day, etc.) as the response variable. When the response variables was binomial (e.g. chick survival), we instead modeled a generalised linear model with a binomial error distribution. In all cases, we then performed Tukey’s Tests to compare the years 2009 and/or 2010 individually with all other years. Tukey’s test allow pairwise group comparison while reducing type-1 errors caused by repeated tests^38^.

The number of incubating pairs in mid-December, the air temperature in winter, as well as the climatic variables in February (when foraging data collection occurred), were inspected visually for anomalies (i.e. extreme values). Any other period that seemed post-hoc to be of importance to the 2009 or 2010 breeding seasons were investigated for climatic anomalies.

### Supplementary Information

Supplementary Information.

## Data Availability

The data associated with the study has been deposited in Dryad and is available at 10.5061/dryad.t76hdr84c.
