# Author Correction: Investigating two consecutive catastrophic breeding seasons in a large king penguin colony

**DOI:** 10.1038/s41598-023-43280-x

**Published:** 2023-10-04

**Authors:** Émile Brisson‑Curadeau, Annette Scheffer, Phil Trathan, Fabien Roquet, Cédric Cotté, Karine Delord, Christophe Barbraud, Kyle Elliott, Charles‑André Bost

**Affiliations:** 1 UMR 7372‑CNRS, Centre d’Études Biologiques de Chizé, La Rochelle University, Villiers‑en‑Bois, France; 2 Natural Resource Sciences, McGill University, Sainte‑Anne‑de‑Bellevue, QC Canada; 3 OKEANOS Centre, University of the Azores, 9901‑862 Horta, Portugal; 4 British Antarctic Survey, High Cross, Madingley Road, Cambridge, CB3 0ET UK; 5 National Oceanography Centre, Waterfront Campus European Way, Southampton, SO14 3ZH UK; 6 Department of Marine Sciences, University of Gothenburg, Gothenburg, Sweden; 7 Laboratoire d’Océanographie et du Climat: Expérimentations et Approches Numériques (LOCEAN‑IPSL), CNRS, IRD, MNHN, Sorbonne Université, Paris, France

Correction to: *Scientific Reports* 10.1038/s41598-023-40123-7, puplished online 10 August 2023

The original version of this Article contained errors in the legends of Figure 1 and Figure 3. The legends of these Figures were inadvertently switched.

The legend of Figure 1:

“Foraging effort (left) and foraging success (right) variables during incubation. The year 2010 is highlighted in red. Asterisks are displayed above years that are significantly different from 2009 (Tukey’s Test).”

now reads:

“Chick survival throughout the winter. Results for the 2009 breeding season is in blue and results for the 2010 breeding season is in red. For a given period, survival rates that are significantly different than either 2009 or 2010 are represented with an asterisk (*), those that are significantly different than both years are represented with an eight-spoke star (⁕).”

The legend of Figure 3:

“Chick survival throughout the winter. Results for the 2009 breeding season is in blue and results for the 2010 breeding season is in red. For a given period, survival rates that are significantly different than either 2009 or 2010 are represented with an asterisk (*), those that are significantly different than both years are represented with an eight-spoke star (⁕).”

now reads:

“Foraging effort (left) and foraging success (right) variables during incubation. The year 2010 is highlighted in red. Asterisks are displayed above years that are significantly different from 2009 (Tukey’s Test).”

The original Article has been corrected.

