# Supplementary material for: Investigating two consecutive catastrophic breeding seasons in a large king penguin colony
Source: Sci Rep. 2023 Aug 10;13:12967. doi: 10.1038/s41598-023-40123-7 (PMC10415367; doi:10.1038/s41598-023-40123-7)
Supplement: Supplementary file 1 — Supplementary Information. [file 41598_2023_40123_MOESM1_ESM.docx]

**Supplementary Material**

Table S1: Sample size of loggers deployed during incubation

| Year | Number of GPS/ARGOS equipped | Number of depth-loggers equipped |
| --- | --- | --- |
| 1998 | 4 | 0 |
| 1999 | 0 | 0 |
| 2000 | 0 | 0 |
| 2001 | 4 | 4 |
| 2002 | 0 | 0 |
| 2003 | 0 | 0 |
| 2004 | 0 | 0 |
| 2005 | 0 | 0 |
| 2006 | 3 | 3 |
| 2007 | 2 | 2 |
| 2008 | 3 | 2 |
| 2009 | 0 | 0 |
| 2010 | 8 | 2 |
| 2011 | 0 | 7 |
| 2012 | 0 | 0 |
| 2013 | 0 | 0 |
| 2014 | 12 | 7 |
| 2015 | 2 | 3 |
| 2016 | 0 | 0 |
| 2017 | 0 | 0 |
| 2018 | 0 | 0 |
| 2019 | 0 | 0 |
| 2020 | 0 | 0 |
| 2021 | 0 | 0 |
| 2022 | 5 | 7 |

Table S2. Reasons for differences between sample size of the different metrics and number of equipped individuals

| Scenario | Reason |
| --- | --- |
| *Egg abandon rate sample size being higher than the number of equipped individuals* | - **The nests of non-equipped individuals were monitored in 2010 and 2011 (see methods)** - **The nests of individuals equipped with loggers not used in this study (e.g. camera-loggers) were monitored and included in the study** |
| *Egg abandon rate sample size being lower than the number of equipped individuals* | - **Nest failure was sometimes unreported** |
| *Trip length sample size being higher than the number of equipped individuals* | - **The trip lengths of individuals equipped with loggers not used in this study (e.g. camera-loggers) were monitored and included in the study** |
| *Mass gained/day sample size being lower than the number of equipped individuals* | - **To weigh returning equipped penguins, the individuals had to be captured before their arrival at the nest. If penguins returned to the nest unnoticed, the logger was retrieved quickly while the bird was still incubating, to avoid disturbance at the nest. Hence, the mass could not be taken in that case.** |

Table S3: Sample size of loggers deployed during chick-rearing

| Year | Number of GPS/ARGOS equipped | Number of depth-loggers equipped |
| --- | --- | --- |
| 1998 | 17 | 9 |
| 1999 | 6 | 4 |
| 2000 | 8 | 9 |
| 2001 | 3 | 4 |
| 2002 | 9 | 8 |
| 2003 | 8 | 6 |
| 2004 | 8 | 7 |
| 2005 | 4 | 5 |
| 2006 | 7 | 6 |
| 2007 | 4 | 5 |
| 2008 | 2 | 2 |
| 2009 | 4 | 13 |
| 2010 | 0 | 0 |
| 2011 | 6 | 1 |
| 2012 | 0 | 5 |
| 2013 | 6 | 6 |
| 2014 | 6 | 1 |
| 2015 | 3 | 4 |
| 2016 | 11 | 8 |
| 2017 | 7 | 9 |
| 2018 | 6 | 10 |
| 2019 | 4 | 6 |
| 2020 | 8 | 10 |
| 2021 | 11 | 16 |
| 2022 | 13 | 19 |


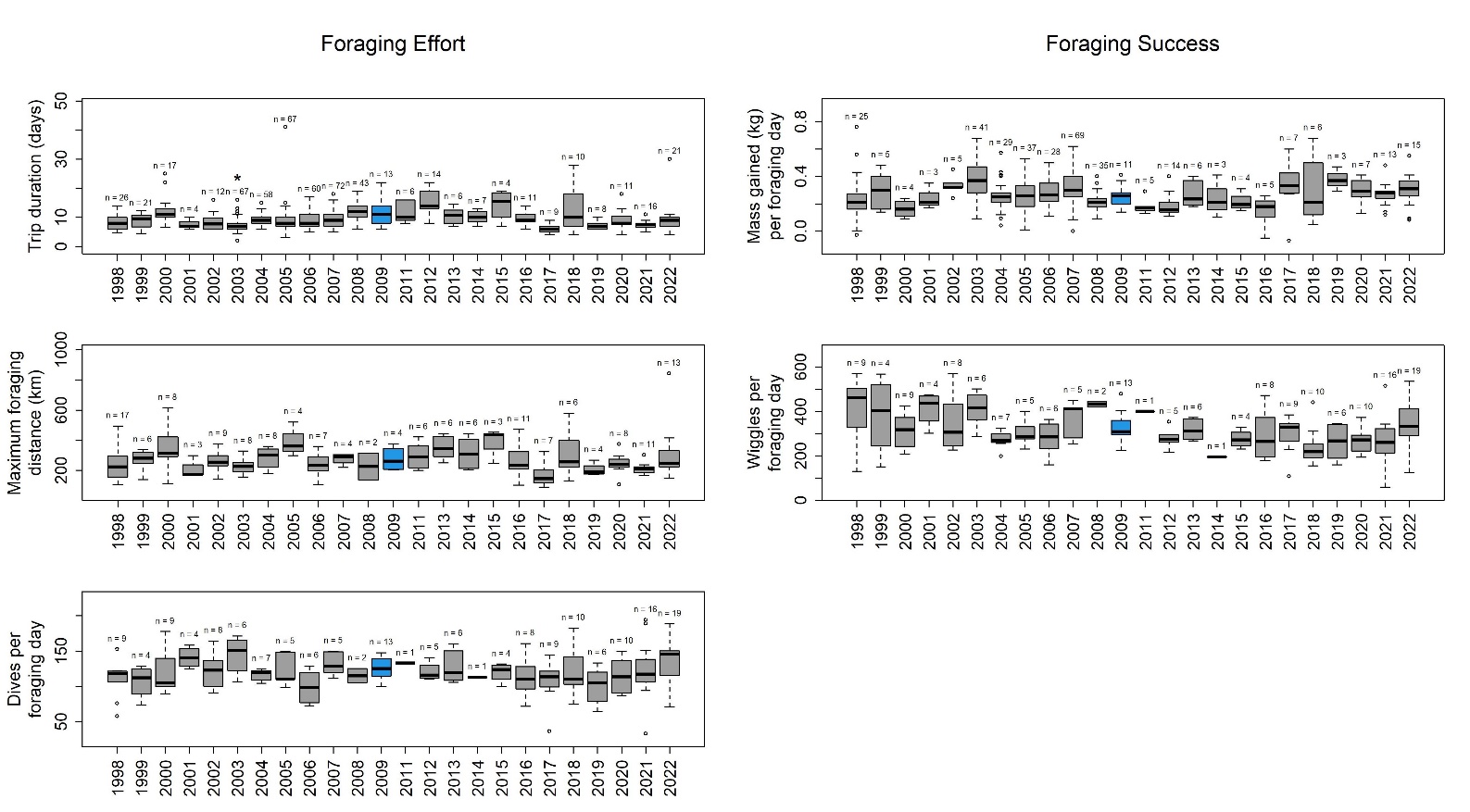


Figure S1: Foraging effort (left) and foraging success (right) variables during chick-rearing. The year 2009 is highlighted in red. Asterisks are displayed above years that are significantly different from 2009 (Tukey’s Test).


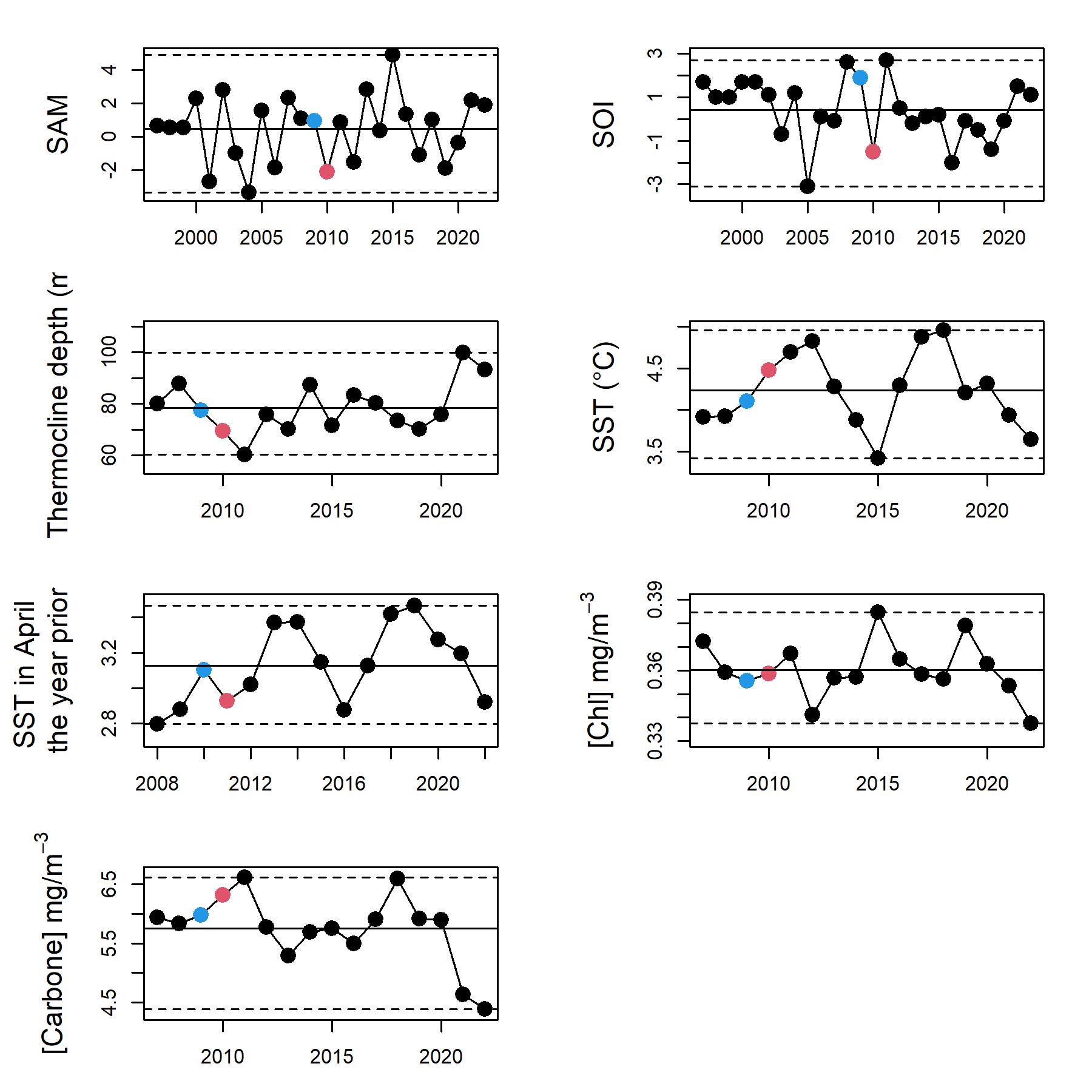


Fig S2: Environmental variables in the foraging zone during February 2009 (blue) and 2010 (red) in comparison with other years from the dataset (black). Maximum and minimum values are represented by a dotted line. Sea ice was not shown, as it is absent from the foraging zone in summer.


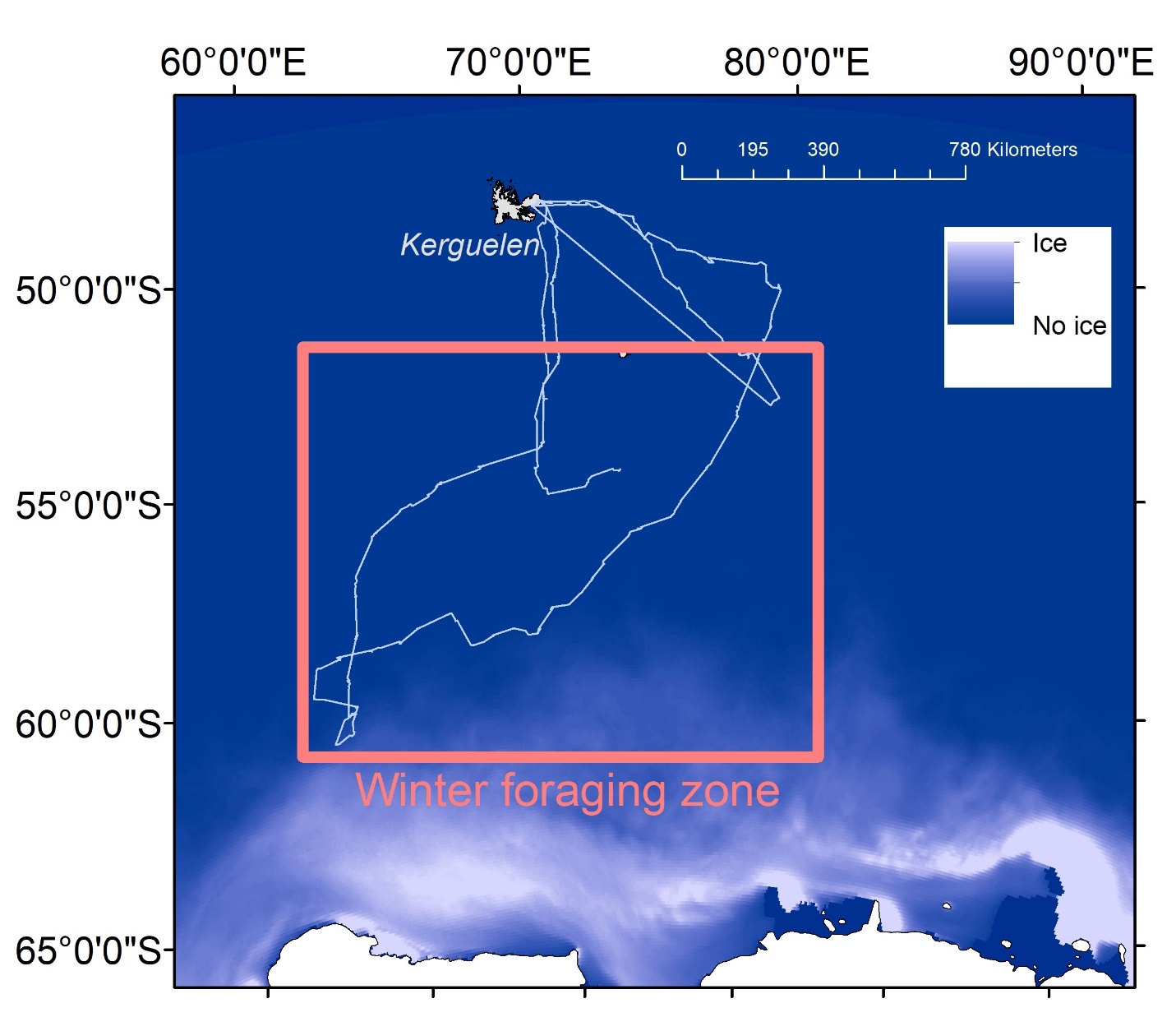


Figure S3: Winter foraging location based on three king penguins equipped with ARGOS Splash tags from Wildlife Computers (Redmont, US). These results are expected to be published in a separate paper in 2024. Map created using ArcGIS Pro 3.1. Country contour lines downloaded from <http://tapiquen-sig.jimdo.com> (Carlos Efraín Porto Tapiquén. Orogénesis Soluciones Geográficas. Porlamar, Venezuela 2015. Based on shapes from Enviromental Systems Research Institute. Free Distribution).
